# Lumbar Spine Injuries in Sports: Review of the Literature and Current Treatment Recommendations

**DOI:** 10.1186/s40798-019-0199-7

**Published:** 2019-06-24

**Authors:** Jacob R. Ball, Colin B. Harris, Jonathan Lee, Michael J. Vives

**Affiliations:** Department of Orthopaedics, Rutgers New Jersey Medical School, 140 Bergen St., ACC D1610, Newark, NJ 07103 USA

**Keywords:** Lumbar, Spine, Athlete, Injury, Sport

## Abstract

Low back pain is one of the most prevalent complaints of athletes at all levels of competition. The purpose of this literature review is to provide an overview of sport-specific injuries and treatment outcomes that can be used by healthcare providers to better recognize injury patterns and treatment options for different groups of athletes. To our knowledge, no prior comprehensive review of lumbar spine injuries in sports is currently available in the literature, and it is essential that healthcare providers understand the sport-specific injury patterns and treatment guidelines for athletes presenting with low back pain following an athletic injury. Injury mechanisms were found to vary significantly by sport, although some broad recommendations can be made with regards to optimal treatment for these injuries and return to play. Additionally, it was found that certain treatments were more beneficial and resulted in higher rates of return to play depending on the specific sport of the injured athlete. Healthcare providers need to be aware of the different injury patterns seen in specific sports in order to properly evaluate and treat these injuries. Furthermore, an individualized treatment plan needs to be selected in a sport-specific context in order to meet the needs of the athlete in the short and long term.

## Key Points

- Lumbar spine injuries are common in athletes.
- Different injury types are seen more frequently in specific sports.
- Treatment options need to be considered in a sport-specific context for the best outcome.

## Background

An estimated 10–15% of all athletes are expected to experience low back pain [1]. Sports such as football and dancing, which place increased stress on the lumbar spine, are believed to have higher rates of low back pain compared to less physically demanding activities [2, 3]. The repetitive flexion, extension, and axial load type movements that athletes place on their spine contributes to their low back pain even though they often possess superior strength and flexibility when compared to the general public [4]. Numerous studies have shown multiple injury patterns in the lumbar spine, demonstrating the increased stresses that elite athletes place on the lower back.

The purpose of this review is to evaluate different sports and the lumbar spine injuries associated with them. Furthermore, this review will evaluate treatment options in a sports specific manner to assess optimal treatment for the athlete with a lumbar spinal injury.

## Main Text

### General Considerations

**Fig. 1:**
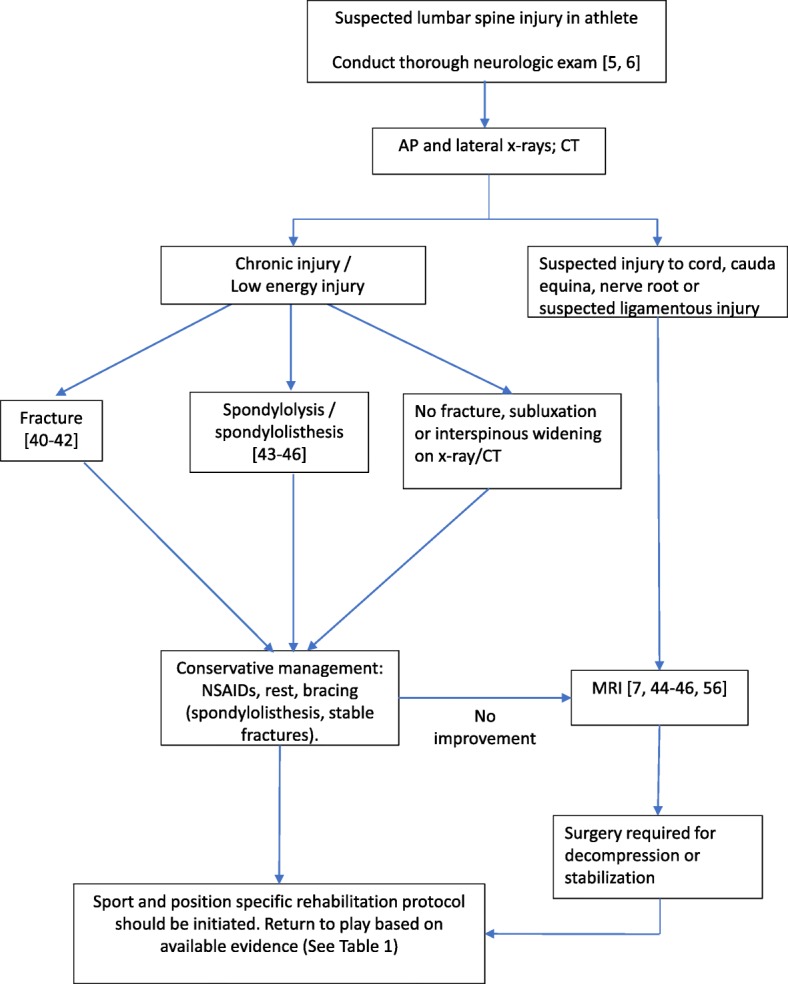
Treatment algorithm for the athlete with a suspected lumbar spine injury

Lumbar spine injuries that occur during play require proper on-field treatment and management to prevent serious complications. Most authors focus on the cervical spine when discussing on-field back injuries, but the general principles can also be applied to the lumbar spine. The most important step in managing on-field injuries is developing an appropriate protocol that specifies the medical equipment needed at every sporting event, the person responsible for evaluating the injured player, and who will contact emergency medical services [5, 6]. The athlete requires a focused musculoskeletal and neurologic exam, but it is essential to minimize spinal movement to prevent further injury [5, 6]. As opposed to cervical spine injuries, logrolls should be avoided when transferring the patient to a spine board [5]. The most important prognostic factor is the time it takes for the athlete to be taken to a healthcare center that is properly equipped for spinal injuries [5] (Fig. 1).

The first steps to evaluate an athlete who presents with low back pain involve a thorough history and physical examination in order to elicit the injury mechanism, identify any neurologic deficits or signs of more serious injury, and to direct the physician to the appropriate workup. Pain due to spondylolysis and facet (posterior element) injuries is reproducible with extension, whereas discogenic pain is reproduced with flexion. A thorough neurological examination is mandatory, including motor, reflex, and sensory testing in addition to provocative tests such as the Spurling’s test and straight leg raise (Lasegue’s) test. When radicular pain or neurological deficits are present, MRI is necessary to detect spinal cord and nerve involvement. For lower back pain, lacking neurological signs, upright AP, and lateral X-rays are generally the initial study of choice but may be non-diagnostic for several common conditions. For pars stress reactions, MRI is useful when plain radiographs including AP, lateral, and oblique views are negative and has the added benefit of not exposing patients to ionizing radiation as seen in CT with SPECT imaging [7]. CT is recommended to assess for fracture, anterolisthesis, or alignment abnormalities in cases of acute trauma and is the first line imaging modality to assess for bony detail of a pars defect or to follow the healing of the pars with conservative treatment. The muscular or ligamentous strain should be suspected when imaging studies are negative for anatomical abnormalities, and before returning to play a brief course of rest and anti-inflammatory medications followed by strength and flexibility training is indicated. The following provides an overview of sport specific injuries which is summarized in Table 1.

**Table 1:** Sport specific lumbar spine injuries and treatment outcomes

Sport	Epidemiology	Prognosis
American football	Up to 30.9% of injuries are lumbar spine related [8]. Twenty-eight percent of lumbar injuries are disc herniations [9]. Avulsions, spondylosis, and strains are also prevalent.	Surgical repair of disc herniation may have return to play advantages [10]. Similar outcomes with microdiscectomy and non-surgical treatments [11].
Ice hockey	Ninety-five percent of players report lumbar pain in final year of play [12]. Thoracolumbar and lumbosacral account for approximately 12% of on-ice spine injuries [13]. Lumbar spondylolysis was diagnosed in 44% of youth ice hockey players complaining of lower back pain [14].	Surgical repair of disc herniations was associated with decreased return to play rates [15, 16]. Return to baseline level of performance during second and third season post-injury [16].
Basketball	10.2% of all injuries involve the lumbar spine [17]. Sprain and strain, lumbar disc degeneration, and lumbar contusions account for 7.9%, 0.9%, and 0.9% of the total injuries respectively [17].	Surgery for disc herniation resulted in decreased performance during the first season after injury. Pre-injury skill returned during second and third season post-surgery [15, 16].
Baseball	89.5% of players report lower back pain during career [18]. 35.1% and 22.8% of players showed signs of L5/S1 or L4/L5 disc degeneration respectively [18].	Hitters and infielders had faster return to play time with nonsurgical interventions whereas there was no difference for pitchers [19].
Soccer (European football)	76.6% of players report low back pain during career [18]. Three percent of injuries occurring in soccer are lumbar spine related [20]. Strains, sprains, spondylosis, and fractures occur in soccer. The most serious injuries are often the result of contact with another player which can lead to fracture.	Fractures resulted in the longest recovery time followed by bony and soft tissue injuries. Repetitive wear and tear type injuries also occur and tend to persist.
Dance	The lumbar spine is the second most commonly injured site [21]. Spondylolysis is the most common injury type seen [22]. The combination of repetitive hyperflexion and poor technique contribute to injury [23]. Males are prone to injury due to lifts they perform [21].	Forty-six percent of injured dancers had to limit up to half of the activity and 5% needed to limit more than half of activity [24].
Gymnastics	Evidence of disc degeneration is as prevalent as 75% in elite athletes [25]. Studies demonstrate an 11% incidence rate of spondylolysis [26].	In general, these injuries are well managed non-surgically, but there is not currently data specific to gymnasts.
Skiing and snowboarding	Lumbar spine is the most common site injured, includes compression, burst, and transverse or spinous process fractures [27, 28]. In snowboarders, injury is associated with failed jumps and the subsequent axial loads. The mechanism of injury in skiers is associated with downhill falls forward at high velocities [27, 29, 30].	Spinal cord injuries are more commonly seen with cervical spine injuries, and less common with thoracic and lumbar spine injuries [31]. Most injuries are managed non-operatively.

### American Football

Football is a very popular sport in the USA, with many players becoming involved in the early teenage years and participating in high-level practice and competition by ages 15–18. Although cervical spinal cord injuries sustained during football are high profile due to their potentially catastrophic nature and have received the greatest media attention, lumbar spine injuries are common (30.9% of injuries in one study) and can lead to considerable morbidity and lost playing time [8, 9]. Disc herniations account for about 28% of lumbar spine injuries in football, with the majority located at L5-S1 and L4-L5 [9]. Mechanism of injury is not well understood but is related to blocking and tackling, with offensive and defensive linemen being the most commonly injured players by position. Non-contact injuries also account for about 20% of lumbar spine injuries, likely from avulsion due to sudden changes in the direction [8]. Another common injury sustained by football athletes is spondylolysis, secondary to extension and rotation forces in the lumbar spine. These injuries generally respond well to bracing in the adolescent population but often require direct pars repair or fusion in adults in order to expedite return to play.

### Ice Hockey

While ice hockey is a popular sport in the USA, few studies have investigated the prevalence and mechanism of lower back pain in the sport. One small study found that 95% of ice hockey players reported lumbar spine pain in their final year of play [12]. However, MRI changes in thoracolumbar vertebrae in ice hockey players were studied over a 15-year interval and it was found that new abnormalities from baseline were infrequent [32]. A more common mode of injury was that existing vertebral abnormalities continued to degenerate over the 15-year period [32]. The baseline characteristics of these players were that they had a median age of 24 and had been playing hockey since the age of 10 [32]. These inclusion criteria and the results of the study led the investigators to conclude that most of the injuries sustained to the thoracolumbar spine occurred during the growth spurt phase of their adolescence and it is these injuries that persist throughout their career [32]. A study of Canadian ice hockey leagues found that for injuries that occur during play, 82% were cervical, 7.3% were thoracolumbar, 5.1% were thoracic, and 4.8% were lumbosacral [13]. Further investigation revealed that being checked from behind was the most common cause of injury at 35% [13]. Another small study followed a single elite youth ice hockey program over a period of 9 seasons and found that of the players who complained of lower back pain, 44% were diagnosed with lumbar spondylolysis [14]. Furthermore, 73% of spondylolysis occurred on the shooting side of the player which suggests that the directional rotation of the spine may lead to specific injury patterns [14]. Future studies investigating specifically the causes of lumbar spinal injury in ice hockey would be beneficial to better understand injury and prevention.

### Basketball

Injuries in basketball are common due to the increasingly physical nature of the game. Studies have not specifically evaluated the mechanisms of lumbar spine injuries in basketball, but it is likely related to a combination of torsion, loading, and trauma. In a study that tracked all injuries in the National Basketball Association (NBA) over a 17 year period, researchers found 10.2% of all injuries were in the lumbar spine [17]. Lumbar spine injuries were second only to ankle injuries, which accounted for 14.7% of total injuries [17]. On further analysis of the total injuries in the NBA over the 17 year period, lumbar sprain and strain accounted for 7.9% of injuries, lumbar disc degeneration accounted for 0.9% of injuries, and lumbosacral contusion accounted for 0.9% of injuries [17]. Even though lumbar disc degeneration only resulted in 0.9% of total injuries, it accounted for 3.6% of the total games missed, which indicates how serious these injuries are when they occur [17]. Following surgical repair of a lumbar disc herniation, multiple studies have shown that the return to play rate and the games played the following season both decrease [15, 33]. Furthermore, for the players that were able to return the next season, their player efficiency ratings were significantly reduced [33]. Interestingly, games played and player efficiency ratings for the second and third season post-injury returned to their preinjury level, indicating that players do well in the long term from these surgeries [33].

### Baseball

Baseball is a physically demanding sport due to the kinetic chain needed for successful throwing and batting motion. Unfortunately, few studies have directly examined the mechanisms of baseball injuries, but there are some data on epidemiology and injury specific treatment outcomes. One study examining the Major League Baseball (MLB) injury list between 2002 and 2008 found that 11.7% of the players had sustained either a spine or core injury [34]. Interestingly, both pitchers and fielders experienced spine and core injuries at a similar rate during this time period [34]. In another study that evaluated college athletes in Japan, T2 MRI revealed that 59.7% of baseball players showed signs of disc degeneration [18]. Furthermore, 35.1% of baseball players showed signs of disc degeneration at L5/S1 and an additional 22.8% of players showed disc degeneration at L4/L5, suggesting that the lumbar spine is most susceptible to injury [18]. Lastly, 89.5% of the baseball players studied reported having low back pain at some point during their life [18]. In a study assessing lumbar disc herniation and treatment, it was found that players who underwent surgery had a significantly longer recovery period than players who received non-operative treatment (8.7 vs 3.6 months) [19]. Interestingly, the treatment plan and the return to play time varied depending on player position [19]. For pitchers, there was no significant difference in the return to play time for surgical versus nonsurgical management, but hitters and infielders had a significantly shorter return to play time with nonsurgical treatment [19]. While this was a small study, the data suggest that there are also worse performance outcomes in the surgical group compared to the nonsurgical group [19]. Future studies should focus on specific mechanisms of lumbar spine injury in baseball as well as a wider variety of injury types and treatments.

### Soccer (European Football)

Soccer is the world’s most popular sport, and low back injuries are not uncommon. A study by Hangai et al. found that in college athletes in Japan, the lifetime incidence of low back pain in soccer players was 76.6% compared with 53.5% of non-athletes [18]. One study that took place in the UK followed more than 12,000 highly competitive youth soccer players for five complete seasons and found that out of the 10,225 injuries that occurred, only 310 (3%) were related to the lumbar spine [20]. Of the 310 lumbar spine injuries, 49.4% were classified as low back pain, 15.2% as sprains, and 4.2% as spondylolysis [20]. Furthermore, lower back injuries recorded by anatomical site resulted in the lumbar region accounting for 44.5% followed by erector spinae and quadratus lumborum at 11.9% and 5.8% respectively [20]. With respect to recovery time from injury, fractures took the longest with a median of 148.5 days followed by bony tissue and soft tissue injuries at 15.5 and 13 days respectively [20]. Not surprisingly, contact with other players was found to cause significantly more injuries than non-contact play [20]. Another study that identified 137 consecutive cases of spondylolysis in New York City youth athletes found that the largest percentage (19%) of these children were soccer players [35]. The authors stressed the importance of understanding which sports are most prevalent in a given region because even though spondylolysis may be rare in soccer players, the volume of players in a region makes certain injuries more prevalent [35]. Lastly, in a study that included 70 former professional soccer players, they found that the incidence of osteophytes in the lumbar spine was significantly increased when compared to control groups [36]. This finding suggests that through competitive play, the mechanical forces on the lumbar spine ultimately result in osteophyte development [36]. Given the global popularity of soccer and the lumbar pathology experienced by players throughout their career and in retirement, future studies are needed to better understand the mechanisms of injury to create a safer environment [18–20, 35, 36].

### Dance

While participation in dance is less than in some of the sports discussed above, it is technically demanding and physically challenging which increases the risk of injury. Past studies have suggested that the body positions required for dance cause dangerous hyperflexion of the lumbar spine, but more recent literature trends towards improper technique as the main cause of injury [23]. In a study that followed professional ballet dancers for 10 years, researchers found that the injury incidence per dancer per year was 1.1, which suggests that dancers can expect at least one injury every year [23]. The foot and ankle were the most frequently injured area (38%), followed by the lumbar spine (20%); however, the lumbar strain was the single most common diagnosis made during this period [21]. The authors of the study claimed that male dancers have higher rates of lumbar strain because the lifts they perform cause intense lower back stress [21]. Another study that specifically evaluated youth dancers found that lumbar spine injuries made up 11.7% of all injuries occurring during dance [22]. Furthermore, the most common skeletal injury to occur in this population was spondylolysis [22]. There have also been case reports of pediatric ballet dancers who presented with bilateral pedicle fractures without spondylolysis, which demonstrates the wide differential diagnosis needed when assessing a dancer presenting with lower back pain [37]. For dancers who experienced an episode of lower back pain, one study reported that 46% of the participants had to limit up to half of their dance activity with another 5% limiting more than half of their activity [24]. Due to the nature of dance, the injury rate remains high and the lumbar spine is especially vulnerable as evidenced by previous studies.

### Gymnastics

While the posterior column injuries, specifically spondylolysis and spondylolisthesis, are the most written about, other studies have noted anterior and middle column injuries in gymnasts to include disc herniation, compression fractures, disc degeneration, and Schmorl’s nodes [25, 38]. In a study assessing magnetic resonance imaging in 33 competitive female gymnasts without regard to the presence or absence of back pain, evidence of degenerative disc disease was found in 24%, with increasing rates of degeneration as age and competitive level increased to 63% of 8 national or Olympic gymnasts with an average age of 25.7 years [38]. Similarly, in a study of 24 elite male gymnasts with an average of 23 years of age, there was a 75% prevalence of disc degeneration seen on MRI compared with 31% in control populations [25].

Lumbar isthmic spondylolysis in gymnasts is well described. These injuries were noted to be chronic in nature, without a specific inciting event, as a result of repetitive hyperextension in back walkovers and rotation in dismounts, vaults, and flips. A classic study involving lumbar radiographs of 100 competitive gymnasts with ages ranging from 6 to 24 found an 11% incidence of lumbar spondylolysis [26]. This represents a four times higher incidence of pars interarticularis defects than the 2.3% found in the general female Caucasian population. Furthermore, 6 of these 11 had first-degree spondylolisthesis of L5 on S1. With continued vertebral slippage above 50%, the gymnast’s ability is hampered by a vertical sacrum, decreased flexibility, and hamstring tightness.

### Skiing and Snowboarding

Traumatic brain injury and vertebral injuries represent the most common injuries among severely injured skiers and snowboarders [39, 40]. The mechanism of injury is different between snowboarders and skiers [27, 29, 30]. In snowboarders, injury is associated with failed jumps and the subsequent axial loads. The mechanism of injury in skiers is associated with downhill falls forward at high velocities.

Multiple studies have investigated spinal injuries in skiers and snowboarders. In a 6-year retrospective review of a tertiary trauma center in Switzerland, the most common site of severe spinal injury for skiers and snowboarders combined was the lumbar spine [28]. Out of 148 total spinal fractures identified in 73 patients, there were 55 severe lumbar spine injuries with a breakdown of 16 burst fractures, 15 compression fractures, 22 transverse process fractures, and 1 incident of traumatic spondylolysis. Severe spinal injury occurred most commonly in skiers, with 17 requiring intervention for the lumbar spine. In another retrospective study, over the course of 11 years, 41 skiers and snowboarders with spinal fracture or dislocations were identified with 12 cervical, 25 thoracic, and 20 lumbar injuries [41]. This represents an overall incidence of spinal trauma of 1 per 100,000 skier days. Within the lumbar injuries, 17 were compression fractures (all with height loss < 25%) and 3 were transverse process fractures; no burst fractures were noted. None of the patients required surgery. A study based in Japan which evaluated 13,490 cases of ski- or snowboard-related injury identified an incidence of 5.73 per 100,000 visits for snowboarders and 0.69 per 100,000 visits for skiers [27]. Across skiers and snowboarders, lumbar injuries were the most common at 64.8% and 69.4% respectively. The most common fracture patterns were anterior compression and transverse process fractures. Spinal injuries occurred more commonly in snowboarders, with risk factors including beginners with simple falls, and intermediate or experts jumping. In a study evaluating both thoracic and lumbar injuries only in Colorado, out of 146 fractures, the majority of fractures were simple compression fractures (*n* = 81), as well as burst fractures (*n* = 26) and transverse and spinous process fractures (*n* = 32) [42]. Only 1 patient in the compression and burst fractures group required surgical stabilization. A study evaluating an inpatient database of skier and snowboarder injuries found that skiers were more likely to injure the cervical spine and snowboarders were more likely to injure the lumbar spine [31].

Many of the studies pointed out that injuries commonly occurred at multiple levels. Spinal cord injuries are more commonly seen with cervical spine injuries, and less common with thoracic and lumbar spine injuries [31]. There have been no studies regarding soft-tissue injuries such as disc herniations, facet capsule tears, and muscle strains. In summary, lumbar spine fractures are the most common type of spinal injuries in skier and snowboarders.

### Other Sports

Repetitive flexion, extension, and torsional stress of the lumbar spine also occur in sports beyond those detailed in this review. However, a thorough literature search uncovered insufficient data to allow conclusions to be drawn about the prevalence or pattern of lumbar injuries that can be discerned in other sports.

### Treatment guidelines

#### Fractures

Minor fractures are the most common type of lumbar spine fracture seen in athletes and are due to repetitive activity or low-energy impact. Major fractures are much more serious and often lead to functional or neurological deficits and result from high-energy mechanisms. The severity of the fracture can be categorized by which segments of the spine are involved, based on the three-column spine model [43]. Minor fractures are defined as involving the pars interarticularis, articular process, transverse process, or the spinous process but do not result in instability [43]. Major fractures tend to be unstable and include compression fractures, burst fractures, seat-belt-type injuries, or fracture-dislocations [43, 44]. The thoracolumbar junction is susceptible to injury during high-energy impact as the more stable thoracic spine transitions to the mobile lumbar spine [45].

Major fractures which are unstable enough to necessitate surgery are rarely seen in sports, given the high energy required to cause such an injury. Most lumbar fractures seen in sports can be treated non-operatively [45]. In terms of medical care, symptomatic treatment such as rest, NSAIDs, muscle relaxants, and the use of bracing is sufficient for fractures limited to one column of the spine such as the spinous process, transverse process, or the vertebral body [45]. Facet fractures are also known to occur in isolation but usually only require bracing if unilateral and have no additional fractures present. Non-surgical techniques may improve return to play rates, but more data are needed to better guide treatment.

#### Spondylolysis and Spondylolisthesis

It has been reported that up to 47% of young athletes with low back pain will ultimately be diagnosed with spondylolysis [46]. One study using MRI found that 39.7% of children younger than 19 who complained of LBP for more than 2 weeks had spondylolysis [47]. Of the children who presented with LBP, 9.3% of elementary students, 59.3% of junior high schoolers, and 31.5% of higher schoolers were confirmed with MRI to have spondylolysis [47]. The repetitive extension and twisting of the lumbar spine required in soccer, gymnastics, and football are the reasons why these sports are most commonly associated with spondylolysis. The most common presentation is a patient with low back pain that is exacerbated by spinal extension but rarely has neurological involvement. More serious injuries such as spondylolisthesis can also present similarly, but neurological deficits may be seen most often in the L5 distribution. Definitive diagnosis begins with PA and lateral upright radiographs of the lumbar spine followed by MRI due to its superior capability to detect pars edema (stress fracture) which is missed by plain radiographs and CT [48]. Use of oblique views and CT with SPECT imaging is less common due to the radiation exposure to the athlete. Interestingly though, small studies have found that MRI alone would not have been sufficient for a successful diagnosis of spondylolysis in the pediatric population [49]. There is some evidence to suggest that it is important to incorporate CT and plain radiographs in the screening protocol for LBP [49]. Bilateral pars defects, which occur the majority of the time (85%), are likely to progress to spondylolisthesis. Conservative treatment options for acute pars defects or stress reactions include a combination of stabilization and physical therapy such as using a full-time Boston brace for 8–12 weeks, a graduated exercise program, and isometric core and hamstring exercises [50, 51]. One study using CT imaging separated spondylolysis patients into categories of early, progressive without high signal intensity, progressive with high signal intensity, and terminal defects [52]. Using a hard brace, the investigators found that 94% of patients with early spondylolysis responded well to this conservative treatment [52]. The efficacy for the progressive groups was significantly lower, with 64% for progressive without signal intensity, 27% for progressive with signal intensity, and 0% for terminal spondylolysis, demonstrating that the effectiveness of conservative treatment varies with the progression of the injury [52]. Furthermore, the use of bracing itself is questionable, with some studies showing no significant difference in outcomes when no brace is used during conservative therapy [53]. Surgery is considered in athletes who fail to improve or still have symptoms after bracing, continue to experience pain and inability to return to sport for longer than 6 months, or have worsening spondylolisthesis to greater than 50% slip [51, 54]. For athletes with spondylolisthesis or disc degeneration, anterior and posterior fusion is often required; however, if there is no pathology of the disc, pars repair and debridement of the fibrous defect is sufficient [51]. When deciding on the best surgical technique, there are multiple considerations. Posterior lumbar interbody fusion (PLIF) has been associated with more consistent results than posterolateral fusion (PLF), but PLF has been found to be associated with greater patient satisfaction [55, 56]. In addition, anterior interbody fusion was associated with less morbidity than posterolateral intertransverse process fusion but had similar patient outcomes [57]. In terms of recovery, athletes can expect to return to play after 6–12 months but if lumbar fusion was performed, this may be a career-ending option and return to contact sports is not recommended by many practitioners [58].

#### Lumbar Disc Herniation

Sports that require repetitive flexion and compression such as football, ice hockey, basketball, and soccer are often associated with lumbar disc herniation. Common signs of lumbar disc herniation are lower back pain with progression to radicular pain and occasionally neurologic deficits, depending on the severity of injury. In the National Football League (NFL), approximately 28% of spinal injuries are due to lumbar disc herniation [9]. Linemen were the most susceptible to this type of injury because of repetitive spine extension due to blocking as well as a weight training program [9]. The most common site of disc herniation was between L4 and L5, followed by L5 and S1 [9]. A thorough history that assesses for signs of urinary or bowel incontinence is essential to rule out cauda equina syndrome which requires urgent attention [59]. Additionally, a complete neurological exam is needed to identify signs of dysfunction that may require further investigation. Positive nerve root tension signs are an indication for imaging of the lumbar spine which includes upright radiographs and MRI [59]. In one large study of 342 professional athletes, 82% of players were able to return to play after sustaining a lumbar disc herniation, which indicates a favorable prognosis [10]. The mainstay therapy is conservative management including early activity and core strengthening with additional sport-specific activity as symptoms improve. While epidural corticosteroid injections may have a role in shortening the duration of treatment, there is not enough evidence currently available for this to be conclusive [60]. Athletes who undergo laminotomy and disc fragment excision have a return to play rate that is consistent with patients treated with non-surgical options and 81% of these patients will play for an additional 3.3 years [10, 61]. However, the beneficial or harmful effects of surgery are variable, depending on the sport [10]. For NFL players, it was found that surgery resulted in a significant increase in games and years played when compared to conservative therapy [10]. Furthermore, there may also be a significant return to play advantage for NFL players who undergo surgical intervention for lumbar disc herniation [62]. However, for MLB players, surgery resulted in fewer games and years played when compared to conservative therapy, which demonstrates the need to tailor therapies to individual athletes [10]. Data regarding new minimally invasive techniques is not yet conclusive but decreased soft tissue manipulation could lead to faster recovery and return to play times for athletes [11]. Studies have found similar results when comparing microdiscectomy to nonsurgical treatments in elite NFL players [11]. Recovery from lumbar discectomy in elite athletes has been reported to range from 2.8 to 8.7 months with an average career length of 2.6 to 4.8 years [63].

#### Return-to-Play after Lumbar Injuries

Return to play after sustaining one of the lumbar spine injuries listed in Table 2 requires resolution of symptoms, full range of motion of the lumbar spine, and the ability to perform sports-specific movements without pain. Both surgical and non-surgical treatment for lumbar disc herniation in athletes have a comparably high recovery and return to play rate. One study found that at 3, 6, and 12 months post-surgery, 50%, 72%, and 84% of patients were eligible to return to play, respectively [64]. However, it was found that only 38 to 65% of players who underwent surgical treatment for a lumbar disc herniation were able to return to the same level of play as prior to the injury [65]. Additionally, one meta-analysis has noted that the age at which a player undergoes surgery is an important factor in determining career length post-operatively, but the relationship is sport-dependent [65]. In the National Hockey League, regardless of surgical versus non-surgical treatment, players with a lumbar disc herniation played in 56.2 games per season pre-injury versus 39.0 games per season post-injury [66]. This suggests that sustaining an injury is a more critical factor in a player’s career than the type of treatment that is selected. Interestingly though, when compared to healthy controls, it was found that players in the National Basketball Association who underwent surgical intervention for lumbar disc herniation had a more normal career length than players who underwent non-surgical interventions [16]. Different physical requirements imposed upon athletes by different sports may explain the career length differences in the surgical versus non-surgical interventions. For the conservative treatment of lumbar spondylolysis, some investigators recommend between 4 and 12 weeks of rest and immobilization [51]. Conservative treatment is associated with a return to play rate of 80%, which is why it is often tried prior to surgical intervention [67]. At 6–12 months after a pars repair, return to play at pre-injury level is possible, but after fusion for spondylolysis and spondylolisthesis there is a less predictable course of returning to contact sports. [68]. An important consideration for return to play that is specific to fusion for spondylolysis and spondylolisthesis is whether radiographs show bony union [58]. Players who undergo lumbar fusion may require up to 12 months of rehabilitation prior to returning to play.

**Table 2:** Common lumbar spine injuries in sports and their treatments

Injury type	Treatment
Fractures	Minor fractures are best treated with rest, NSAIDs, muscle relaxants, and bracing.
Spondylolysis and spondylolisthesis	Full-time Boston brace is the first line treatment for early spondylolysis [52]. Physical therapy alone that includes exercise programs and stretches is acceptable [53]. Posterior lumbar interbody fusion, posterolateral fusion, or anterior interbody fusion are used as surgical management [56, 57].
Disc herniation	Early activity and core strengthening are first-line treatments for conservative management. Laminotomy and disc fragment excision have an 81% return to play rate [10, 61]. NFL players benefited from undergoing surgical correction, but MLB players were less well off than players who underwent nonsurgical therapy [10, 62].

## Conclusions

Lumbar spine injuries can range from minor strains to high-energy fractures, and each of these requires their own set of treatments and return to play guidelines. Additionally, team physicians need to have algorithms that can rapidly assess on-the-field injuries that require vastly different interventions depending on type and severity. The recovery process and ultimately return-to-play by athletes largely depends on the type of injury sustained as well as the athlete’s progression back to pre-injury level of activity. Furthermore, and most importantly, the risk of further injury always needs to be paramount when deciding on appropriateness of return to play.

## Data Availability

Not applicable.

## Abbreviations

AP: Anteroposterior
CT: Computerized tomography
LBP: Low back pain
MRI: Magnetic resonance imaging
PLF: Posterolateral fusion
PLIF: Posterior lumbar interbody fusion
SPECT: Single-photon emission computerized tomography
